# Understanding and managing acute encephalitis

**DOI:** 10.12688/f1000research.20634.1

**Published:** 2020-01-29

**Authors:** Rashmi Kumar

**Affiliations:** 1 Department of Pediatrics, King George's Medical University, Lucknow, India

**Keywords:** Acute encephalitis syndrome, Japanese encephalitis, autoimmune encephalitis

## Abstract

Encephalitis is an important cause of morbidity, mortality, and permanent neurologic sequelae  globally. Causes are diverse and include viral and non-viral infections of the brain as well as autoimmune processes. In the West, the autoimmune encephalitides are now more common than any single infectious cause, but, in Asia, infectious causes are still more common. In 2006, the World Health Organization coined the term “acute encephalitis syndrome”, which simply means acute onset of fever with convulsions or altered consciousness or both. In 2013, the International Encephalitis Consortium set criteria for diagnosis of encephalitis on basis of clinical and laboratory features.

The most important infectious cause in the West is herpes simplex virus, but globally Japanese encephalitis (JE) remains the single largest cause. Etiologic diagnosis is difficult because of the large number of agents that can cause encephalitis. Also, the responsible virus may be detectable only in the brain and is either absent or transiently found in blood or cerebrospinal fluid (CSF). Virological diagnosis is complex, expensive, and time-consuming. Different centres could make their own algorithms for investigation in accordance with the local etiologic scenarios. Magnetic resonance imaging (MRI) and electroencephalography are specific for few agents. Clinically, severity may vary widely. A severe case may manifest with fever, convulsions, coma, neurologic deficits, and death.

Autoimmune encephalitis (AIE)  includes two major categories: (i) classic paraneoplastic limbic encephalitis (LE) with autoantibodies against intracellular neuronal antigens (Eg: Hu and Ma2) and (ii) new-type AIE with autoantibodies to neuronal surface or synaptic antigens (Eg: anti-N-methyl-D-aspartate receptor). AIE has prominent psychiatric manifestations: psychosis, aggression, mutism, memory loss, euphoria, or fear. Seizures, cognitive decline, coma, and abnormal movements are common. Symptoms may fluctuate rapidly.

Treatment is largely supportive. Specific treatment is available for herpesvirus group and non-viral infections. Various forms of immunotherapy are used for AIE.

Encephalitis is an important cause of morbidity, mortality, and permanent neurologic disability in both adults and children. The term “encephalitis” literally means inflammation of part or all of the “encephalon” or brain parenchyma. Globally, invasion by a pathogen causing direct neuronal injury is the most common cause of encephalitis. Direct injury by pathogen invasion may result in acute, subacute, or chronic manifestations. Sometimes, the inflammation is not due to invasion but is an indirect immunologically mediated injury.

Non-inflammatory diffuse dysfunction of the cerebrum is termed as “encephalopathy” and common causes include metabolic, toxic, or ischemic disorders. The term encephalopathy is also used for altered mentation due to any cause. Encephalopathy can also be acute or chronic.

Encephalitis and meningitis are overlapping syndromes. Pathologically, both viral and non-viral invasions of the brain cause some degree of both meningeal and parenchymal inflammation ^1^. Therefore, many clinicians prefer the term “meningoencephalitis”. Clinically, there is a spectrum of manifestations, but by and large two distinct patterns are seen ^2^. When the illness is associated with prominent sensorial alterations, the clinical syndrome is said to be “encephalitis”; when meningeal irritation is prominent, it is called “meningitis”. The term “aseptic meningitis” is used for a self-limited meningitic presentation and little or no sensorial alteration ^3^.

The magnitude of the problem of encephalitis is difficult to assess because of a lack of uniform definitions and study populations. Worldwide the prevalence varies. In the US, there were 73 hospitalizations due to encephalitis per 100,000 population (95% confidence interval 7.1–7.6) ^4^ from 2000 to 2010. In Ontario, Canada, between 2002 and 2013, the crude incidence of all-cause encephalitis was estimated to be about 4.3 cases per 100,000 persons per year ^5^. The problem is further compounded by the fact that a wide variety of central nervous system disorders may mimic encephalitis. Infectious encephalitis can occur in epidemic, endemic, or sporadic form. In 2006, the World Health Organization coined the term “acute encephalitis syndrome” (AES) for the purpose of surveillance of JE. This is defined as “an illness in a person of any age and at any time of the year characterized by acute onset of fever with alteration of consciousness, stupor, coma and/or new onset of convulsions excluding simple febrile convulsions” ^6^. Basically, this refers to acute illness with fever along with altered sensorium or convulsions or both. This type of presentation can have a very wide etiology. Table 1 shows the etiology of AES according to pathophysiology ^7^. The prevalence and etiology of AES may vary from region to region because of geographical differences and also from time to time. In India, AES constitutes a public health problem.

**Table 1.** Etiology of acute encephalitis syndrome.

I. Viral agents that are known to cause encephalitis Arboviruses, togaviruses, and alphaviruses • Western equine encephalitis virus • Eastern equine encephalitis virus • Venezuelan equine encephalitis virus Flaviviruses (mosquito-borne) • Japanese encephalitis virus • St Louis encephalitis virus • West Nile virus • Murray Valley encephalitis virus • Non-arthropod-borne togavirus – rubella virus Bunyaviruses • California encephalitis virus Reoviruses • Colorado tick fever encephalitis virus Herpesviruses • Herpes simplex 1 and 2 • Varicella zoster virus • Epstein–Barr virus • Cytomegalovirus • Human herpesvirus – 6 • B virus Enteroviruses • Polioviruses • Coxsackie viruses • Echoviruses • Enteroviruses 70 and 71 Orthomyxoviruses • Influenza viruses Paramyxoviruses • Measlesvirus • Mumpsvirus • Parainfluenza viruses • Nipah virus Adenoviruses Parvoviruses Rhabdoviruses	○ Tuberculousmeningitis ○ Mycoplasma pneumoniae ○ Listeria monocytogenes ○ Spirochetes: syphilis, Leptospirosis, ○ Lyme disease ○ Brucellosis ○ Legionella ○ Salmonella typhi ○ Cat scratch disease (Bartonellosis) Fungi: Cryptococcus, histoplasma, aspergillus, mucormycosis, candida, coccidiomycosis Protozoa: naeglaria, acanthameba, *Toxoplasma gondii* Metazoa: Trichinosis, echinococcus, cysticercus, schistosoma III.Non-infectious inflammation of brain • Acute disseminated encephalomyelitis • Antibody-associated encephalitis • Collagen vascular disorders IV.Infectious encephalopathy • Cerebral malaria • Shigella encephalopathy • Dengue encephalopathy • Sepsis syndrome • Enteric encephalopathy V. Structural causes of coma with associated fever due to another cause • Tumor • Vascular event • Head injury • Other space occupying lesion VI.Functional causes of coma with associated fever due to another cause • Electrolyte encephalopathy • Reye’s syndrome • Diabetic coma • Uremic coma • Hepatic coma • Inborn error of metabolism • Chemicals • Toxins • Hypertensive encephalopathy
II.Non-viral agents • Rickettsia: Rocky mountain spotted fever, endemic and epidemictyphus, Coxiellaburnetti, Ehrlichiosis, scrub typhus • Bacterial: ○ Pyogenic (bacterial) meningitis	

The table is based on data from 7.

In 2013, the International Encephalitis Consortium (IEC) suggested consensus criteria for case definition of encephalitis or encephalopathy of presumed infectious or autoimmune etiology ^8^. Neurologic dysfunction in the form of altered mental status lasting for over 24 hours without an alternative cause must be present. Minor criteria that must be present (two for possible and at least three for probable or confirmed) include “fever ≥38°C within 72 hours, seizures, new focal neurologic findings, CSF pleocytosis (≥5 white blood cells [WBCs]/μL), neuroimaging with brain parenchymal changes or electroencephalogram (EEG) consistent with encephalitis. Definite cases require pathologic confirmation on brain biopsy, evidence of infection with a microorganism associated with encephalitis, or laboratory evidence of an autoimmune condition associated with encephalitis” ^8^. Thus, the IEC case definition does not differentiate infectious from post- or non-infectious processes ^8^.

## Acute encephalitis syndrome etiology

The most important cause is infection—both viral and non-viral. Viral agents include primary neurotropic viruses such as arboviruses, herpesviruses, and rabies virus as well as other “incidental” nervous system pathogens such as entero, orthomyxo, paramyxo, and adenoviruses, which primarily cause disease elsewhere in the body and involve the central nervous system only occasionally ^7^. Worldwide, JE is the single largest cause of viral encephalitis (VE) ^9^. Non-viral brain infectious agents include bacteria, mycoplasma, rickettsiae, protozoa, metazoa, and fungi. Non-infectious brain inflammation such as in AIE, acute disseminated encephalomyelitis (ADEM), and collagen vascular disorders is another group. In infectious encephalopathies, there is a systemic infection causing altered sensorium or convulsions without invasion of the brain, thus presenting with the clinical syndrome of AES ^7^. Lastly, the patient may have a functional coma (due to metabolic causes, drugs, toxins, chemicals, and so on) or a structural coma (due to tumors, space-occupying lesions, and vascular events). If there is also concurrent fever due to any other cause, such patients would present as AES ^7^.

In high-income Western countries, the incidence of AIE surpasses that of any single infectious cause ^10^. The most common sporadic infectious encephalitisis is herpes simplex virus encephalitis (HSVE) ^11^. The California Encephalitis Project enrolled 1570 patients with suspected encephalitis over a 7-year period (1998–2005): “A core battery of tests for 16 agents was performed. In addition, selective testing for other agents were performed on the basis of clinical and epidemiologic features. Only 16% of patients had a *confirmed or probable* etiologic agent identified, of which 69% were viral, 20% bacterial, 7% prion, 3% parasitic and 1% fungal. An additional 13% had *possible* etiologies identified. In this group there were many agents not hitherto implicated as a cause of encephalitis. Autoimmune etiology was found in 8% which made it more common than any single infectious agent. The remaining 63% had no etiology identified” ^10^.

In most Asian countries, infectious causes are still far more common. In a study on VE in Chinese children, the etiology was confirmed in 52.5% of patients. The most common pathogen was human enterovirus (EV) (27.7%). The etiology of viral meningitis was identified in 42.8% of cases; the leading pathogen again was human EV (37.7%) ^12^. In India, JE is probably the commonest form and occurs in epidemics, especially in the south and east. Rabies is another form of VE which poses a public health problem. In the Gorakhpur division of eastern Uttar Pradesh, JE was held to be the main agent responsible for AES. There was a severe epidemic of JE in this region in 2005 ^13^, after which the Government of India imported the Chinese live attenuated vaccine (SA-14-14-2 strain), which was administered to children in affected districts. The proportion of AES caused by JE declined in recent years but AES itself did not decrease ^14^. Over the last 5 to 7 years, a lot of literature has emerged from various parts of India, implicating scrub typhus (caused by *Orientia tsutsugamushi*) meningoencephalitis as an important cause of AES here ^15– 18^. Nipah virus, a paramyxovirus known to cause severe encephalitis in Malaysian pig farmers, caused an outbreak of encephalitis in the southern state of Kerela ^19, 20^. Dengue infection was known to produce neurologic manifestations, including encephalopathy, but dengue is now also recognized as a cause of VE due to actual viral invasion of the brain ^21, 22^. In Muzaffarpur, Bihar, outbreaks of acute neurologic illness occurred which are believed to be due to toxins, namely hypoglycin and methylene cyclopropylglycine present in litchi fruit ^23^. A recent study from Lucknow, described trend of infectious encephalitis after immunization for JE was incorporated. Evidence of JE was found in 8.3%, dengue 7.8%, EVs 0.4%, HSV 0.8%, varicella zoster 0.4%, scrub typhus meningoencephalitis in 31.8%, *Haemophilus influenza* in 0.97%, and *Streptococcus pmeumoniae* in 0.94% ^24^.

So it is clear that a wide variety of infectious and non-infectious etiologies are associated with AES/encephalitis. However, in both Western industrialized nations and developing countries, the cause in more than half of cases remains unexplained despite extensive testing.

## Epidemiology

Several factors such as age, geographical location, season, climate, and host immune status affect the epidemiology of encephalitis. Arboviruses or arthropod-borne viruses have their life cycle in insect vectors and vertebrate animals, occasionally infecting humans who are a “dead end “host ^25^. Different arboviral encephalitides have their own specific geographical distribution depending on the activity of their insect vectors. For example, arboviral encephalitides prevalent in the US include Western equine, eastern equine, Californian, and St. Louis encephalitis. Venezuelan encephalitis is found in South America, and JE in Asia. These encephalitides tend to occur in epidemics or outbreaks. Occasionally, a fresh pathogen, when introduced into a susceptible population, produces an explosive outbreak. HSVE is the commonest sporadic infectious encephalitis in Western countries. It tends to occur worldwide with little seasonal or age and sex predilection ^4^. West Nile encephalitis virus was introduced in the US in the 1990s and the illness is endemic there ^26^. Mumps, measles, and rabies encephalitis have been largely eradicated from many developed countries because of effective vaccination programs.

## Pathogenesis

Though poorly understood for some etiologies, a variety of mechanisms contribute to encephalitis. Encephalitis may be (i) infectious, resulting from direct invasion of the brain, most commonly gray matter by the pathogen, and (ii) immune-mediated, caused by immune-mediated damage (commonly white matter). Within the infectious group, both neurotropic and non-neurotropic (incidental) pathogens can cause encephalitis. Neurotropic viruses can cause viremia, subsequently crossing the blood–brain barrier (for example, arboviruses), or enter the brain by retrograde axonal transport (for example, rabies virus). Neuronal infection causes release of cytokines leading to cytotoxicity, inflammation, and tissue damage ^27^. Another mechanism is vasculitis leading to tissue ischemia as happens with varicella zoster virus ^28^. Michael *et al*. (2015) measured levels of 38 mediators in serum in 78 and CSF in 37 specimens from patients with encephalitis, 17 of whom had HSVE ^29^. The authors found that a pro-inflammatory cytokine response was associated with greater blood–brain barrier permeability, clinical severity, and damage seen on neuroimaging. Higher serum interleukin 1 receptor antagonist (IL-1RA) levels were found in patients with a good outcome ( *P* = 0.004). These investigators therefore suggested that IL-1 antagonists should be investigated as adjunctive treatment in encephalitis ^29^.

AIE includes two major categories: (i) classic paraneoplastic LE associated with autoantibodies against *intracellular neuronal antigens* (for example, Hu and Ma2) and (ii) new-type AIE associated with autoantibodies to neuronal *surface or synaptic antigens*. Paraneoplastic LE results from an immunological response to tumor antigens, which mimic *intracellular* antigens in neurons. These are strongly associated with cancer, and prognosis tends to be poor because of irreversible neuronal killing by these mechanisms. The “new-type” AIE, of which anti-NMDAR induced is the commonest, occurs in association with pathogenic autoantibodies against membrane antigens or synaptic receptors. Binding of autoantibodies to their targets causes neuronal dysfunction, usually reversibly ^30, 31^. An extracellular epitope in the GluN1 subunit of the NMDAR is recognized by these antibodies, which crosslink the NMDARs and promote internalization of the receptors. This decreases receptor density on neuronal surface and results in neuronal dysfunction. This dysfunction is reversible with removal of autoantibodies. Over a dozen new-type autoantibodies have been identified since the discovery of anti-NMDAR antibody by Dalmau *et al*. in 2007 ^32^. The same process is described for AMPAR encephalitis. Other autoantibodies may work through different mechanisms. For example, anti-LGI1 antibodies block binding sites of LGI1. Anti-GABAb R antibodies block the inhibitory effects of baclofen on the spontaneous firing of cultured neurons and influence receptor function ^30^.

Most of these autoantibodies are associated with specific and well characterized symptoms and the detection of these autoantibodies confirms the diagnosis. Direct brain viral infection triggering of immune-mediated disease may also coexist, as illustrated by HSV encephalitis cases with subsequent or concurrent anti-NMDAR antibodies identified ^33^. A recent study from New Delhi on 40 children who tested positive for autoantibodies showed that NMDAR antibodies were the commonest (60%), followed by anti-basal-gangliaantibody (17.5%), anti-GAD (7.5%), anti-Ma (5%), anti-TPO (5%), anti-Yo(2.5%), and anti-CRMP5 (2.5%) ^34^.

## Clinical features

Geographic and seasonal factors, occupation, history of recent travel, contact with animals, and animal bite need to be considered. JE occurs in summer and post-monsoon epidemics in Asia. A setting of immunodeficiency may point to specific agents like cytomegalovirus ^35^.

Clinical manifestations depend on whether the brain parenchyma or meninges are predominantly involved producing an encephalitic or meningitic syndrome, respectively. However, the same agent may produce a predominantly meningitic picture in one and encephalitic picture in another patient ^36^. The severity of manifestations varies widely from mild febrile illness associated with headache to a severe disorder with convulsions, coma, neurologic deficits, and death ^1^. Usually, the onset is abrupt with fever and declining mental status. There may be irritability, agitation, screaming spells, confusion, delirium, drowsiness, stupor, or coma. Headache may be complained of in older children. Typical features include an initial stage of fever, headache, and vomiting lasting for less than a week followed by convulsions, coma, and neurologic deficits with or without signs of meningeal irritation. Severe cases may be associated with a life-threatening rise in intracranial tension, decerebration, or flaccid coma. Typically, this stage lasts for 7 to 10 days after which there is gradual recovery with or without sequelae ^7^.

Examination should include a search for skin rash often seen with enteroviral encephalitis, measles, dengue, varicella zoster, and rickettsiae. Parotitis often occurs with mumps. Mucous membrane lesions like “cold sore”, believed to be a manifestation of herpes simplex infection, have little diagnostic value. Concurrent upper respiratory infection is characteristic of influenza. Patients with rabies may have the characteristic hydrophobia or aerophobia.

Neurologic signs in acute encephalitis do not reliably identify the underlying etiology despite the propensity of certain neurotropic viruses to affect specific focal areas of the nervous system. A constellation of frontotemporal signs with aphasia, personality change, and focal deficits or focal seizures was considered characteristic of HSE. With the availability of newer non-invasive techniques of diagnosis, milder, atypical cases and an “expanded” spectrum of HSE in children with multifocal or diffuse brain involvement have been described ^37, 38^. Rabies may present as an ascending paralysis simulating Guillain–Barré syndrome ^39^. JE is associated with prominent extrapyramidal or basal ganglia signs in the form of rigidity and abnormal movements, especially in the convalescent stage ^40^.

Cerebral malaria presents as acute onset of fever with chills and rigors, bilateral upper motor neuron signs, and multisystem involvement, including acidosis, severe anemia, hepatic, renal, and pulmonary dysfunction ^41^. ADEM is usually a monophasic illness with multifocal upper motor neuron signs along with altered sensorium.

Two distinct clinical syndromes of infectious AES are now recognized in Uttar Pradesh, India. The first is a pure neurologic illness that includes JE and HSV encephalitis. The second is a multisystem syndrome with rash, thrombocytopenia, hepatosplenomegaly, bleeding, deranged liver, and kidney function and such features are seen in dengue, rickettsial infection, enteric fever, leptospirosis, and cerebral malaria ^42^.

In AIE, psychiatric manifestations are common early in the course. These may include psychosis, aggression, inappropriate sexual behaviors, panic attacks, compulsive behaviors, mutism, memory loss, euphoria, or fear. Symptoms may fluctuate rapidly. In addition, seizures, cognitive decline, coma, and abnormal movements are common ^43, 44^. Clinical differentiation of HSE and AIE may be difficult as psychiatric manifestations and extrapyramidal movements may be seen in both. MRI, polymerase chain reaction (PCR), and antibody detection in CSF/serum may be needed.

## Investigations

Diagnostic testing should include lumbar puncture, brain MRI, and EEG in all suspected cases of encephalitis. After assessment for contraindications, a lumbar puncture with opening pressure measurement should be performed to obtain CSF for total and differential cell counts, diagnostic testing, protein, and glucose. Typically, CSF in VE has essentially normal glucose, increased protein, and a mild to moderate mononuclear pleocytosis (predominantly lymphocytic but can be neutrophilic early in the course) ^4^. Normal CSF WBC counts of infants are higher than those of adults. A 95th percentile cutoff of not more than 19 WBCs/μL for infants below 1 month of age or not more than 9 WBCs/μL for infants 1 to 2 months of age defines pleocytosis ^4^. On the other hand, infectious encephalitis, particularly with EV (~50%) or human parechovirus (HPeV), is more likely without pleocytosis ^4, 8^. Repeat lumbar puncture should be considered in case of persistent or worsening symptoms.

### Investigations for etiology

A large number of viruses are known to cause encephalitis, but virological diagnosis is complex, expensive, and time-consuming. The responsible virus may be detectable only in the brain itself and is either absent or transiently found in blood or CSF. For these reasons, it is not surprising that etiologic diagnosis is not reached in more than half the cases, even at advanced centers ^10^. Different centers could make their own algorithms for investigation according to the local etiologic scenarios. Timing of sampling is important as PCR tends to be positive early in the illness whereas IgM takes 4 to 7 days to appear. For JE, IgM capture enzyme-linked immunosorbent assay (ELISA) in CSF is the gold standard for diagnosis. PCR for JE genome/DNA in CSF or serum, if positive, confirms the diagnosis. JE PCR positivity is highly variable (9–77.8%) in different studies, and early samples have a higher rate of positivity ^45– 47^. Serum IgM and NS1 antigen are used for diagnosis of dengue encephalitis/encephalopathy. ELISA in serum for detection of rickettsial IgM is used for diagnosis of scrub typhus meningoencephalitis. Multiplex PCR panels for a variety of agents are being developed for etiologic diagnosis of encephalitis. Detection of autoantibodies in CSF or serum confirms a diagnosis of AIE.

MRI of the brain with and without contrast using diffusion, T2-weighted, and fluid-attenuated inversion recovery (FLAIR) sequences is the modality of choice to assess changes consistent with brain parenchymal inflammation. A specific MRI picture may be seen with a few pathogens. Herpes simplex encephalitis classically affects temporal and frontal lobes. Focal edema is seen in the medial aspects of the temporal lobes, orbital surfaces of the frontal lobes, insular cortex, and cingulate gyrus ^48^. JE causes T2 hyperintensities in thalami, basal ganglia, and brain stem ^49^. Temporal lobe changes are seen in a small proportion of JE also ^50, 51^. Imaging findings in patients with AIE can be normal or quite variable. Recognizing characteristic findings within limbic structures can alert clinicians to the potential diagnosis.

EEG should be used to look for evidence of encephalopathy, localizing signs, or subclinical seizure activity. Characteristic patterns such as periodic localizing epileptiform discharges are seen in about a third of patients with HSVE ^52^. An extreme delta brush pattern may be seen in patients with AIE ^53^.

## Treatment

A severe case should be managed in an intensive care unit. An active search for treatable causes of AES should continue along with measures to protect the brain from further insult. Management may be divided into supportive and specific.

### Supportive treatment

1. Airways, breathing, and circulation should be maintained. Body temperature should be brought down by use of appropriate antipyretic and cooling methods. Hydration, electrolyte status, and acid–base parameters should be maintained within normal range. It is prudent to use appropriate parenteral antibiotics until a bacterial cause is excluded with certainty.

2. Convulsions are best managed with intravenous anticonvulsants such as phenytoin or valproate which do not depress the sensorium. Tube feeding may be started once the patient is stabilized and convulsions are controlled. Care must be taken to prevent aspiration, and the protocol for management of a comatose patient should be followed. The patient should be placed in a lateral or semiprone position.

3. In the presence of features of raised intracranial pressure, mannitol infusion (0.25 to 1.0 mg/kg every 4 to 6 hours) or intravenous furosemide may be needed. Hypertonic saline (3%) in a dose of 0.1 to 1 mL/kg per hour in order to maintain serum sodium between 145 and 155 mEq/L is another option. Hyperventilation to keep arterial carbon dioxide (CO _2_) tension between 25 and 30 mm Hg may also be used to combat raised intracranial tension. When facilities are available, monitoring of intracranial tension is useful. In the case of rapidly increasing intracranial tension with clinical deterioration unresponsive to medical treatment, surgical decompression can be life-saving ^54^.

4. Gastric hemorrhage (stress ulcer) is a common event ^13^ and should be managed with antihistamines, antacids, ranitidine, and (if necessary) a blood transfusion.

5. The role of steroids in acute infectious encephalitis is debatable. There are theoretical arguments for and against their use. On the one hand, a life-threatening rise in intracranial tension may be relieved by steroids; on the other hand, there is a risk of flaring-up of viral infection with steroids. A study that evaluated high-dose dexamethasone in JE found no benefit of steroid therapy ^55^.

### Specific therapy

Among the viral agents, specific therapy is recommended in encephalitis due to the herpes group of viruses. Acyclovir in a dose of 10 mg/kg administered as an intravenous infusion over 1 hour every 8 hours for 14 days (21 days in immuno-compromised cases) is indicated in HSVE ^56^. The success of antiviral therapy depends on early institution of therapy. In many centers in the West, in the absence of an epidemic, acyclovir is started as soon as VE is suspected clinically, regardless of whether any evidence of localization is present. In the Indian context, no hard criteria can be laid down since the relative importance of HSVE in different regions of the country is not really known. It seems reasonable to start acyclovir in non-seasonal cases, especially if focal features are present or neuroimaging studies reveal temporal lobe involvement. The toxicity and side effects of acyclovir include bone marrow suppression, vomiting and hypotension after intravenous administration, and (occasionally) non-oliguric renal failure in dehydrated patients ^56^. Confusion, hallucinations, seizures, and coma are rare. Blood counts and relevant biochemical parameters should be closely monitored. Caution should be exercised in the presence of renal impairment as 80% of the drug is excreted unchanged in the urine ^57^. Relapses may occur in as high as 5% of cases ^54^. Resistance to acyclovir is rare (about 0.5% in immunocompetent patients) ^58^. Foscarnet is used for acyclovir-resistant HSE ^59^. Acyclovir is also recommended for varicella zoster encephalitis, and ganciclovir is an alternative drug. A combination of ganciclovir and foscarnet is recommended for cytomegaloviral encephalitis ^59^. Pleconaril is an investigational agent for enteroviral infections, which acts by inhibiting viral attachment and uncoating ^60^. Oseltamivir can be considered for influenza (H1N1) encephalitis/encephalopathy ^59^. Trials with interferon alpha and nasogastric ribavirin in JE in children have revealed no benefit ^61, 62^. The tetracycline drug minocycline is a known neuroprotective agent with antiviral properties and excellent penetration into CSF. A trial of nasogastric minocycline in AES in Lucknow revealed modest benefit in terms of both cumulative mortality at 3 months from onset and neurologic sequelae ^63^. Intravenous azithromycin or oral minocycline/doxycycline is currently recommended for rickettsial meningoencephalitis ^64^.Treatment of AIE is immunosuppression through high-dose methylprednisolone pulse therapy, intravenous immunoglobulin, plasmapheresis, rituximab, and azathioprine ^31^.
